# Determinants of Sprint Ability Change During Maturation in Developing Children

**DOI:** 10.1002/ejsc.70133

**Published:** 2026-01-30

**Authors:** Masamichi Okudaira, Ryosuke Takeda, Tetsuya Hirono, Taichi Nishikawa, Shun Kunugi, Kaito Igawa, Saeko Ueda, Yukiko Mita, Kohei Watanabe

**Affiliations:** ^1^ Faculty of Education Iwate University Iwate Japan; ^2^ Laboratory of Neuromuscular Biomechanics School of Health and Sport Sciences Chukyo University Aichi Japan; ^3^ Human Health Sciences Graduate School of Medicine Kyoto University Kyoto Japan; ^4^ Ritsummeikan Global Innovation Research Organization Ritsumeikan University Shiga Japan; ^5^ Center for General Education Aichi Institute of Technology Aichi Japan; ^6^ Graduate School of Health and Sport Sciences Chukyo University Toyota Japan; ^7^ Food and Nutritional Environment Human Life and Environment Kinjo Gakuin University Aichi Japan; ^8^ Department of Human Nutrition School of Life Studies Sugiyama Jogakuen University Aichi Japan

**Keywords:** breakpoint, development, leg length, muscle strength, muscle thickness

## Abstract

This study aimed to clarify changes in sprint ability and spatiotemporal variables with maturation in children and to identify key determinants from anthropometric and muscle strength perspectives. Ninety‐eight young soccer players aged 7.6–17.9 years underwent a 30‐m sprint test, anthropometric measurements, muscle thickness (MT) and maximal voluntary contraction (MVC). Maturity offset (MO) was calculated as the difference between the chronological age and estimated age at peak height velocity (PHV). Breakpoint (BP) in maximal sprint speed (MSS) development was identified at +1.1 years of MO, and the participants were classified into pre‐ and post‐BP groups. A significant correlation was found between MSS and MO in the pre‐BP group, but was no longer observed in the post‐BP group. Although step length (SL) was positively correlated with MO in the pre‐BP group, this correlation was not observed and step frequency (SF) showed a positive correlation in the post‐BP group. Multiple regression analysis revealed that in the pre‐BP group, leg length (LL) was the only significant predictor of MSS, primarily by influencing SL. By contrast, in the post‐BP group, MVC and MT emerged as significant predictors of MSS, mainly by influencing SF. In conclusion, this study identified a key developmental BP at +1.1 years of MO and demonstrated a shift in the determinants of sprinting ability from reliance on anthropometric growth before this point to a dominance of neuromuscular factors.

## Highlights

- A developmental breakpoint in maximal sprint speed was observed at +1.1 years relative to the maturity offset.
- Before this breakpoint, sprint ability was primarily associated with leg length, which was the only significant predictor of step length and maximal sprint speed.
- After this breakpoint, the determinants shifted to neuromuscular factors: muscle strength and thickness became significant predictors of maximal sprint speed, mainly by influencing step frequency.

## 1 Introduction

Sprinting abilities develop significantly between childhood and adulthood (Schepens et al. 1998). Sprint speed is determined by the product of step length (SL) and step frequency (SF), making the dynamics of spatiotemporal variables during sprinting a key focus for researchers and practitioners (Hunter et al. 2004; Salo et al. 2011; Nagahara, Matsubayashi, et al. 2014; Nagahara, Naito, et al. 2014). Notably, the increase in SF with age is minimal or nearly negligible, whereas improvements in SL have a substantial impact on sprint performance development (Meyers et al. 2015; Nagahara et al. 2018, 2019). Additionally, the ground contact time (CT) during sprinting does not naturally decrease with age (Nagahara et al. 2018, 2019) and may even increase throughout development (Meyers et al. 2015). Thus, children primarily improve their sprinting ability by increasing SL while maintaining CT and SF during development.

There is a debate regarding the primary factors that determine SL and SF during sprinting (Hunter et al. 2004; Nagahara et al. 2018b). SL is often reported to correlate strongly with anthropometric characteristics such as leg length (LL) and body height (Nagahara et al. 2018b). Another study suggested that muscle strength and power also play key roles in determining SL (Hunter et al. 2004). In contrast, a higher SF is attributed to improvements in neural function, coordination and stiffness characteristics (Meyers et al. 2015; Nagahara and Murata 2020). Despite these findings, there is insufficient research to clarify the factors influencing the development of SL and SF from childhood to adulthood.

Developmental trends in sprint ability and spatiotemporal variables with chronological age have been examined previously (Schepens et al. 1998; Nagahara et al. 2018, 2019). Nagahara, Takai, Haramura, et al. (2018) reported that improvements in sprinting ability tend to stagnate around puberty. However, when assessing changes in physical ability during childhood, it is crucial to consider biological maturity rather than chronological age (Beunen and Malina 1988; Philippaerts et al. 2006). As the timing of the growth spurt varies among individuals, it remains unclear whether the observed stagnation in sprinting ability is a true phenomenon or merely a result of individual differences in maturation, which may mask the significant correlation between chronological age and sprinting ability during certain periods. In recent years, maturity offset (MO), defined as the difference between chronological age and estimated age at peak height velocity (PHV), has become a widely used metric for assessing the biological maturation status of an individual beyond their chronological age (Mirwald et al. 2002; Meyers et al. 2015; Murayama et al. 2023). Several studies have examined the relationship between sprinting ability and MO (Philippaerts et al. 2006; Meyers et al. 2015; Rumpf et al. 2015; Towlson et al. 2018), suggesting distinct phases of accelerated improvement followed by a natural slowing of sprint speed development as an individual reaches physical maturity. This understanding has contributed to the guidelines for youth sprint training (Oliver et al. 2013); however, although such a shift is generally acknowledged, there is no consensus regarding the specific developmental timing of this transition point or breakpoint (BP) (Philippaerts et al. 2006; Meyers et al. 2015; Rumpf et al. 2015; Towlson et al. 2018). Identifying the timing of the BP is important for both training and talent identification. Specifically, this knowledge would allow practitioners to optimise interventions, for instance, by prescribing resisted sprinting or plyometrics to improve SL or by utilising assisted overspeed training to enhance SF, based on an athlete's specific developmental phase (Hunter et al. 2004; Myrvang and van den Tillaar 2024). Additionally, athletes in the accelerated phase have considerable room for natural improvement, whereas those in the decelerated phase have less potential for further gain from maturation alone. This distinction provides valuable information for the practitioners involved in talent identification. Finally, to better understand the developmental trajectory of sprint ability, it is essential to examine how underlying factors, such as LL and muscle strength, change and how these variables correlate with sprint speed and spatiotemporal parameters across different developmental stages.

Therefore, this study aimed to clarify the developmental process of sprint ability and spatiotemporal variables in relation to maturation in children and to identify the determining factors from the perspective of anthropometric factors and muscle strength. We hypothesised that BP could be statistically identified in the developmental trajectory of sprinting ability. Furthermore, we hypothesised that the primary determinants of performance would shift at this BP, and sprint ability would be primarily determined by anthropometric factors (e.g., leg length) influencing SL, whereas after the BP, the key determinants would shift to neuromuscular factors, which would influence both SL and SF, and ultimately, overall sprint speed.

## 2 Material and Methods

### 2.1 Participants and Ethical Approval

This study included 98 male soccer players aged 7.6–17.9 years. Participants were classified as recreationally active trained/developmental, based on the framework proposed by McKay et al. (2022). The typical training frequency varied by age category: U12 players trained three times per week, U15 players trained four times per week and U18 players trained six times per week. Participants' descriptive characteristics (mean ± SD) were as follows: age, 14.0 ± 3.0 years; height, 155.9 ± 15.3 cm and body mass, 48.8 ± 13.8 kg. They had no neurological disorders or orthopaedic problems that could have affected the experimental results. Written informed consent was obtained from parents or legal guardians. The study protocols were approved by the research ethics committee (approval number: 2021‐101) and followed the standards of the *Declaration of Helsinki*. To calculate MO, the PHV age was calculated based on the triple logistic Bock‐Thissen‐du Toit (BTT) model implemented in AUXAL software (Scientific Software International Inc., NC, USA). This model was chosen over more common regression equations (e.g., Mirwald et al. 2002) to provide a more individualised estimation and to mitigate the potential for age‐related biases known to affect such equations, particularly in a cohort with a wide age range. For this model, at least 5 years of annual height data were collected for each participant to estimate the individual age at PHV. MO was calculated as the difference between the chronological age and PHV age. In our participants, it ranged from −4.80 to +7.42 years. As an anthropometric measurement, LL, the distance between the anterior superior iliac spine and the medial malleolus, was measured in the supine position using a tape measure.

### 2.2 Experimental Design

All participants were randomly assigned to either the outdoor or indoor test, and the remaining test was completed after the first test. As an outdoor test protocol, after a 15‐min standardised warm‐up (jogging, dynamic stretch and submaximal short sprint), participants undertook two 30‐m maximal sprints from a 2‐point start, on an outdoor artificial grass pitch. The rest period between sprints was 3–5 min. For the indoor test protocols, participants underwent anthropometric measurements, muscle thickness measurement and maximal voluntary contraction in knee extensor (MVC) measurements.

### 2.3 Sprint Test

The participants started from a stationary 2‐point standing position at 0.5 m before starting a line. Photocells (WT24‐2B410, SICK, Waldkirch, Germany) were set up at 0 and 30 and 2‐m calibration markers were placed on the ground from 20 to 30 m to aid kinematic analysis. Video images (1980 × 1080 pixels) were captured at 240 Hz (LUMIX GH6; Panasonic, Tokyo, Japan). The camera was positioned 30 m from and perpendicular to the 25 m point of the running lane to capture sagittal plane images from touchdown and toe‐off across the last three steps for each athlete from 20 to 30 m. Faster trials enabled the identification of participants suitable for further analysis. The key frames of touchdown and toe‐off were visually identified from the video images using 4× zoom‐in digitising software (Frame‐DIAS6, Q'sfix, Tokyo, Japan). The right and left toes were manually digitised to get the two‐dimensional coordinates at the instant of touchdown. CT and flight time (FT) were calculated by multiplying the number of frames between each event by the reciprocal of the camera frame rate. The SF was calculated as the reciprocal of the step durations, which were determined as the sum of the CT and subsequent FT. The SL was calculated from the scaled horizontal distance between each adjacent toe coordinate at touchdown. Maximal sprint speed (MSS) was calculated as the product of SF and SL.

### 2.4 Muscle Thickness Measurement

Muscle thicknesses (MT) of the vastus lateralis (VL) and vastus intermedius (VI) were measured before MVC measurement. Participants were asked to sit on a chair with their knees and hips at 90°. The reference line for MT measurement was made from the right greater trochanter to the upper lateral edge of the right patella. A longitudinal view of the ultrasound image was captured from the midpoint of the reference line by using an ultrasound B‐mode system (MicrUS EXT‐1H; TELEMED, Vilnius, Lithuania). All MT measurements were performed by one investigator for all participants, and the view of the ultrasound image was carefully checked for consistency. The probe (L12‐5L40S‐3) was initially set perpendicular to the skin, and the image was captured when the brightness of the boundary between the femur and the VI was the highest. Distances from the boundary of the subcutaneous tissue to the deep fascia of the VL and femur were calculated and scaled using imaging software (Image J, National Institutes of Health, Bethesda, MD, USA). The MT was calculated as the sum of VI and VL.

### 2.5 Force Measurement and Analysis

The participants sat on a custom‐made dynamometer (Takei Scientific Instruments Co. Ltd., Niigata, Japan) with their knees and hips at 90° and their ankles fixed to a force transducer (LU100KSE; Kyowa Electronic Instruments, Tokyo, Japan). After a standardised warm‐up session (submaximal isometric contractions of 2 × 50% and 1 × 70% of their subjective effort), the participants performed maximal voluntary isometric contractions of the knee extensor. To elicit maximal contraction, the participants were instructed to gradually increase the knee extension force over 2 s and exert the maximum force for 3 s, with verbal encouragement from the investigators. The force signal was amplified by using a strain amplifier (TSA‐110; Takei Scientific Instruments Co. Ltd., Niigata, Japan), and the maximum force recorded by the system was used as the MVC. MVC measurements were performed twice, with a 2‐min rest interval between trials. The exerted torque was calculated from the product of the extension force and moment arm, which was defined as the distance between the centre of the knee joint and the ankle attachment. The highest MVC value was used for further analyses.

### 2.6 Statistical Analysis

Statistical analyses were performed using R software version 4.3.1 (R Core Team, R Foundation for Statistical Computing, Vienna, Austria). The Shapiro–Wilk test was used to test the normal distribution of all variables. To identify a potential BP in the relationship between the MSS and MO, the Davies test was performed using the *Davies.test* function from the ‘segmented’ package (v 2.0.1) in R. Following the Davies test, segmented regression analysis was conducted using the *segmented* function within the same package R (Muggeo 2003). Based on previous findings (Towlson et al. 2018), the number of BPs was hypothesised to be either one or two, and the model with the lowest Akaike Information Criterion (AIC) value was selected as the optimal model to describe maturational changes in the MSS. The precision of the estimated BP was evaluated using a Wald‐based 95% confidence interval [CI]. For subsequent analyses, the participants were divided into distinct groups based on the BP identified using this procedure. Multiple linear regression analyses were performed to identify the predictors of MSS, SL, SF, CT and FT within each group. In five separate models, each of these variables was treated as the dependent variable and MVC, MT and LL were simultaneously entered as independent variables. Multicollinearity among independent variables was assessed using the variance inflation factor (VIF). Statistical significance was set at *P* < 0.05.

## 3 Results

### 3.1 Maximal Sprint Speed

**FIGURE 1:**
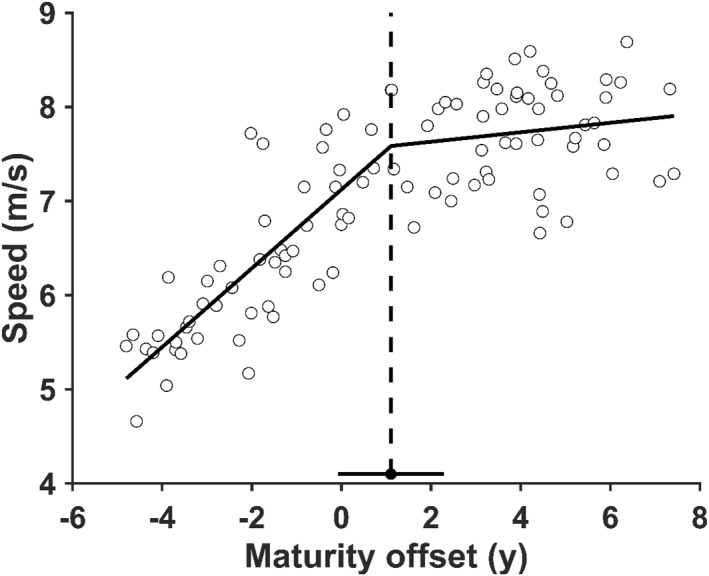
Segmented regression model denoting the breakpoint in the relationship between maximal sprint speed and maturity offset (MO). The vertical dotted line represents +1.1 years of MO and the horizontal line represents 95% CI of the breakpoint.

**FIGURE 2:**
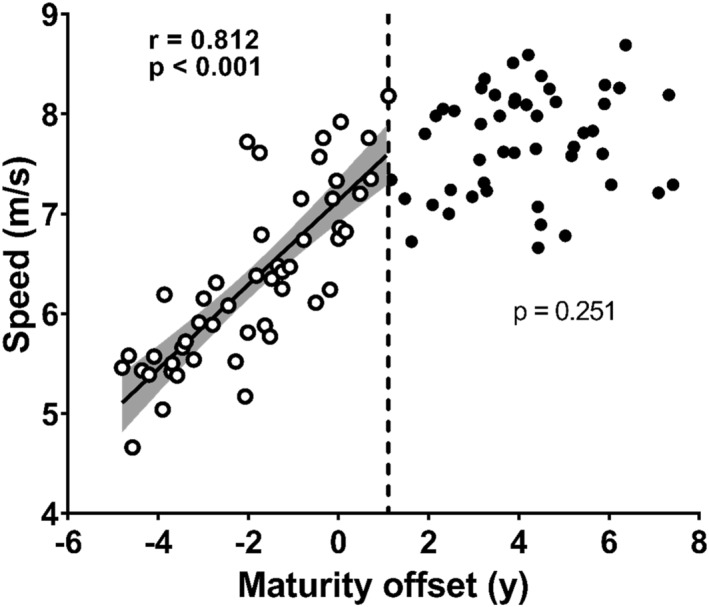
Relationship between maximal sprint speed and maturity offset (MO) in the pre‐BP group (open circles) and the post‐BP group (closed circles). The vertical dotted line represents +1.1 years of MO (breakpoint in maximal sprint speed development). The solid line represents the regression line when correlations were significant within groups, and the shaded area represents the 95% confidence interval.

Segmented regression analyses identified a single statistically significant BP at +1.1 years of MO (95% CI = −0.08–2.29) in a relationship between MSS and MO (Figure 1 and *p* < 0.001). Before BP, a significant positive correlation was observed between the MSS and MO (Figure 2, *r* = 0.812 and *p* < 0.001). However, no significant relationship was observed after BP (Figure 2 and *p* = 0.251). We then divided our participants into two groups: a pre‐BP group and participants younger MO than +1.1 years (*n* = 49). Post‐BP group, participants who showed older MO than +1.1 years (*n* = 49).

### 3.2 Spatiotemporal Variables

**FIGURE 3:**
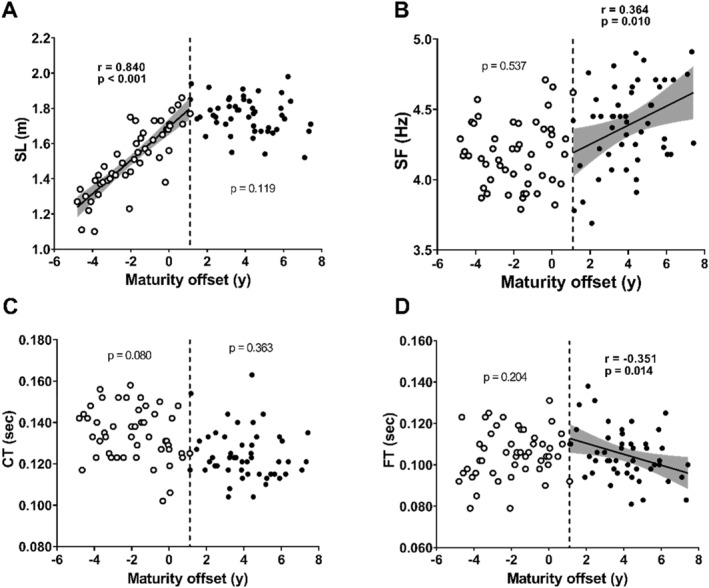
Relationship between spatiotemporal variables and maturity offset (MO) in the pre‐BP group (open circles) and the post‐BP group (closed circles). A, B, C and D represent step length (SL), step frequency (SF), contact time (CT) and flight time (FT). The vertical dotted line represents +1.1 years of MO (breakpoint in maximal sprint speed development). The solid line represents the regression line when correlations were significant within groups, and the shaded area represents the 95% confidence interval.

In the pre‐BP group, a significant positive correlation was observed between SL and MO (Figure 3A, *r* = 0.840 and *p* < 0.001). In contrast, no significant correlation was observed in the post‐BP group (Figure 3A, *p* = 0.119). SF was not significantly correlated with MO in the pre‐BP group (Figure 3B, *p* = 0.537); however, a significant positive correlation was observed between SF and MO in the post‐BP group (Figure 3B, *r* = 0.364 and *p* = 0.010).

Regarding CT, no significant correlation with MO was observed in either the pre‐BP or post‐BP groups (Figure 3C, *p* = 0.080 and 0.363, respectively), although a significant negative correlation was observed when all participants were pooled (Figure 3D, *r* = −0.433 and *p* < 0.001). No significant correlation was observed between FT and MO in the pre‐BP group (Figure 3D and *p* = 0.204); however, a significant negative correlation was found in the post‐BP group (Figure 3D, *r* = −0.351 and *p* = 0.014).

### 3.3 Anthropometric Variable

**FIGURE 4:**
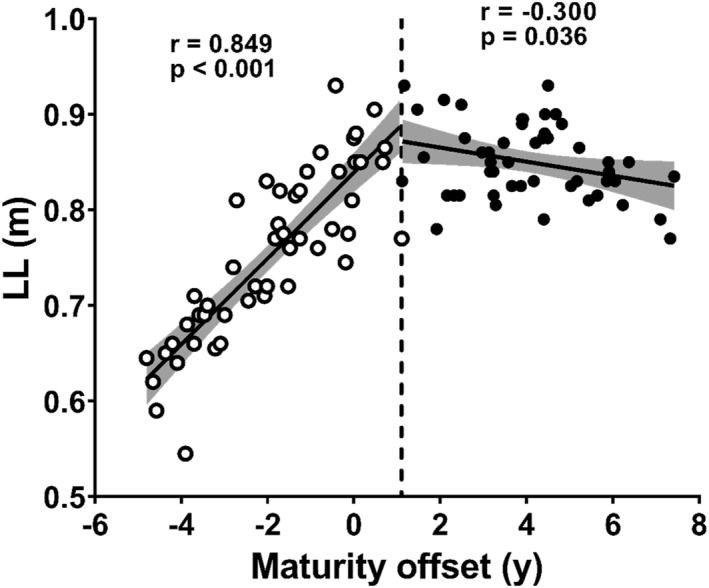
Relationship between leg length (LL) and maturity offset (MO) in the pre‐BP group (open circles) and the post‐BP group (closed circles). The vertical dotted line represents +1.1 years of MO (breakpoint in maximal sprint speed development). The solid line represents the regression line when correlations were significant within groups, and the shaded area represents the 95% confidence interval.

LL showed significant positive correlation with MO in the pre‐BP group (Figure 4, *r* = 0.849 and *p* < 0.001), but a significant negative correlation was found in the post‐BP group (Figure 4, −0.300 and *p* = 0.036).

### 3.4 Maximal Strength and Muscle Thickness

**FIGURE 5:**
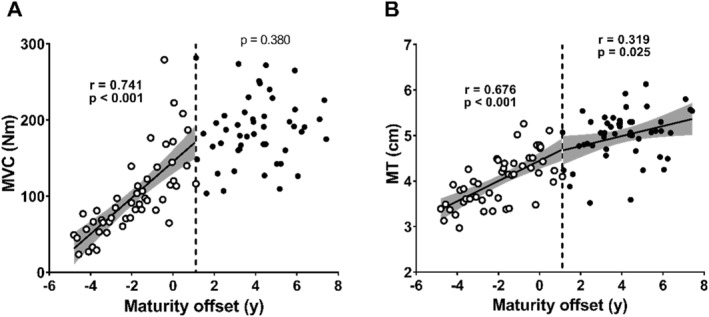
Relationship between (A) maximal voluntary contraction (MVC) in knee extensor and (B) muscle thickness (MT) of the vastus lateralis and vastus intermedius and maturity offset (MO) in the pre‐BP group (open circles) and the post‐BP group (closed circles). The vertical dotted line represents +1.1 years of MO (breakpoint in maximal sprint speed development). The solid line represents the regression line when correlations were significant within groups, and the shaded area represents the 95% confidence interval.

The MVC was significantly and positively correlated with the MO in the pre‐BP group (Figure 5A, *r* = 0.741 and *p* < 0.001), whereas no significant relationship was found in the post‐BP group (Figure 5A and *p* = 0.380). MT demonstrated a significant positive correlation with MO in both the pre (Figure 5B, *r* = 0.670 and *p* < 0.001) and post‐BP groups (Figure 5B, *r* = 0.319 and *p* = 0.025).

### 3.5 Multiple Linear Regression Analysis on Maximal Sprint Speed and Spatiotemporal Variables

For MSS, the regression models were statistically significant for both groups. In the pre‐BP group, the model explained 65.3% of the variance (adjusted *R* ^*2*^ = 0.653), with LL being the only significant positive predictor (Table 1). In contrast, in the post‐BP group, the model explained 26.1% of the variance (adjusted *R* ^*2*^ = 0.261), and the significant predictors shifted to MVC and MT, both of which were positive predictors (Table 1).

**TABLE 1:** Multiple linear regression analysis on maximal sprint speed and spatiotemporal variables.

Dependent variables	Independent variables	Pre‐PB		Post‐PB	
		Standardised regression coefficient	*p* value	Standardised regression coefficient	*p* value
Maximal	MVC	0.262	0.170	**0.368**	**< 0.01** **
Sprint speed	MT	0.008	0.951	**0.358**	**< 0.01** **
	LL	**0.573**	**< 0.01** **	−0.069	0.584
Step length	MVC	−0.153	0.313	0.169	0.210
	MT	−0.026	0.796	−0.01	0.941
	LL	**1.043**	**< 0.001** ***	**0.448**	**< 0.01** **
Step frequency	MVC	**0.926**	**< 0.01** **	**0.225**	**< 0.05** *
	MT	0.081	0.686	**0.377**	**< 0.01** **
	LL	**−0.904**	**< 0.01** **	**−0.461**	**< 0.001** ***
Contact time	MVC	**−0.673**	**< 0.05** *	−0.112	0.392
	MT	0.056	0.788	**−0.370**	**< 0.01** **
	LL	0.405	0.237	**0.274**	**< 0.05** *
Flight time	MVC	−0.318	0.321	−0.176	0.200
	MT	−0.165	0.444	−0.143	0.298
	LL	0.598	0.091	**0.337**	**< 0.05** *

*Note:* Bold values indicate statistically significant standardized regression coefficients and corresponding p‐values (*p < 0.05, **p < 0.01, ***p < 0.001).

Abbreviations: BP, breakpoint; LL, leg length; MT, muscle thickness and MVC, maximal voluntary contraction.

For SL, the models were statistically significant in both groups. LL was the only significant positive predictor of SL in both the pre‐BP group (adjusted *R* ^*2*^ = 0.779) and the post‐BP group (adjusted *R* ^*2*^ = 0.169) (Table 1).

For SF, the models were statistically significant for both groups. In the pre‐BP group, the model explained 12.8% of the variance (adjusted *R* ^*2*^ = 0.128), with MVC and LL as significant positive and negative predictors, respectively (Table 1). In the post‐BP group, the explanatory power of the model increased substantially and accounted for 44.0% of the variance (adjusted *R* ^*2*^ = 0.440). The same predictors were significant, with MVC as a positive predictor and LL as a negative predictor; however, MT also emerged as a significant positive predictor in this group (Table 1).

Regarding CT, the overall model for the pre‐BP group did not reach statistical significance (*p* = 0.129) (Table 1). However, when examining individual predictors within the model, MVC was identified as a significant negative predictor of CT (Table 1). By contrast, the model for the post‐BP group was significant, explaining 21.0% of the variance (adjusted *R* ^*2*^ = 0.210). In the post‐BP model, MT was a significant negative predictor, whereas LL was a significant positive predictor (Table 1).

Finally, for FT, the model for the pre‐BP group was not statistically significant (*p* = 0.331). However, the model for the post‐BP group was significant and explained 13.4% of the variance (adjusted *R* ^*2*^ = 0.134), with LL being the only significant positive predictor (Table 1).

## 4 Discussion

This study aimed to identify the developmental process of sprint ability and spatiotemporal variables and to identify the key determinants of sprint ability in relation to LL and muscle strength. Our findings revealed that the development of MSS in response to MO did not follow a linear trend from −4.80 to +7.42 years of MO and demonstrated a BP at +1.1 years of MO. A significant increase in MSS was observed up to BP, followed by a plateau or a relative stabilisation in MSS improvement compared to the accelerated improvement phase. In the pre‐BP group, LL was the only significant predictor of MSS, primarily influencing SL. By contrast, MVC and MT emerged as significant predictors of MSS in the post‐BP group, mainly influencing SF. Although the nonlinear developmental trend of sprinting ability has been reported previously, the novel finding of this study is the shift in the relative importance of determinants, where the contribution of anthropometric growth diminishes and neuromuscular factors (e.g., muscle strength and thickness) become more predominant. These findings provide valuable insights for athletes and practitioners by offering a foundation for designing effective training programmes and refining talent identification strategies.

### 4.1 Development Trend in the Pre‐BP Period

Children in the pre‐BP group showed a significant positive correlation between MSS and MO (Figure 2), indicating that more mature children demonstrated better MSS up to +1.1 years of MO. There is no clear consensus on the relationship between sprinting ability and maturation (Philippaerts et al. 2006; Meyers et al. 2015; Rumpf et al. 2015; Towlson et al. 2018). For example, Towlson et al. (2018) reported a significant improvement in sprint ability before PHV, followed by stagnation approximately 1.2 years after PHV, which closely aligns with the present results. Conversely, Meyers et al. (2015) found no significant improvement in MSS before PHV but observed substantial development afterwards. Additionally, studies examining sprint ability trends based on chronological age reported stagnation before PHV (Nagahara, Takai, Haramura, et al. 2018), which contrasts with our findings. This discrepancy may be attributed to differences in the developmental patterns of the SL and SF. In the present study, the SL significantly in the pre‐BP group, whereas the SF showed no consistent trend (Figure 3). Previous studies reported a decreasing trend in SF before PHV (Meyers et al. 2015; Nagahara, Takai, Haramura, et al. 2018), which has been linked to factors such as reduced motor coordination (commonly referred to as adolescent awkwardness) (Beunen and Malina 1988; Meyers et al. 2015) and the inability to generate force relative to body mass (Nagahara, Takai, Haramura, et al. 2018). Consequently, the SF tends to stagnate or decline around the PHV, potentially hindering the development of MSS (Meyers et al. 2015; Nagahara, Takai, Haramura, et al. 2018). Therefore, although the increase in SL before +1.1 years after PHV plays a crucial role in MSS improvement, the extent of this improvement may depend on whether the SF declines around the PHV.

### 4.2 Development Trend in the Post‐BP Period

In the post‐BP group, no relationship was identified between MSS and MO (Figure 2), suggesting that beyond 1.1 years after PHV, the direct contribution of maturation to MSS may become less apparent in cross‐sectional observations. This relative stabilisation can be explained by the lack of further increases in SL as no significant relationship was found between SL and MO in the post‐BP group (Figure 3A). SL development is significantly influenced by anthropometric factors such as LL (Hunter et al. 2004). A correlation between LL and MO was observed in the pre‐BP group; however, no such relationship was found in the post‐BP group (Figure 4), which consequently limited further MSS development. However, the deceleration in anthropometric growth alone may not fully account for the MSS stagnation as our data showed that maturational gains in muscle size continued (Figure 5B). This divergence between the trajectory of muscle growth and that of functional performance is attributed to the participants' lack of systematic resistance training. Such training is considered essential for inducing the neuromuscular adaptations required to translate increased muscle mass into functional strength (e.g., MVC) (Stricker et al. 2020; Naimo et al. 2021) and, consequently, MSS.

Although MSS remained relatively stable, a significant correlation was observed between SF and MO in the post‐BP group (Figure 3B). SF is determined by the combination of CT and FT. Although no correlation was observed between CT and MO in post‐BP children, FT showed a significant correlation with MO (Figure 3C and 3D), suggesting that SF beyond 1.1 years after PHV may be driven by a reduction in FT. The present study does not provide direct evidence regarding the mechanisms underlying FT reduction. However, given that shorter FT and higher SF in maximal sprinting are associated with large hip flexion moments (Dorn et al. 2012), it can be hypothesised that maturation‐related reductions in FT are linked to the development of hip flexor muscle strength. However, further research is required to confirm this hypothesis.

### 4.3 Determinants of Maximal Sprint Speed in the Pre‐BP Period

Multiple regression analysis revealed that in the pre‐BP group, both MSS and SL were significantly predicted by LL but not by MVC or MT (Table 1). Because LL is directly associated with contact and take‐off distances (Hunter et al. 2004), this finding suggests that LL is the primary determinant of SL, thereby contributing to MSS in pre‐BP children. Indeed, the high explanatory power of this model (Adjusted *R* ^*2*^ = 0.779 for SL) underscores the dominant contribution of anthropometric growth to SL during this period. The nonsignificant contribution of MVC to these performance outcomes should be interpreted with some caution, given the moderate multicollinearity observed between LL and MVC (VIF = 5.81 and 4.87, respectively). This statistical relationship likely reflects the fact that increases in limb length and muscle strength are concurrent features of development during this period.

Although the MVC was not a direct predictor of MSS or SL, it was a significant predictor of both SF and CT (Table 1). Specifically, greater MVC was associated with higher SF. These results indicate that even during this early developmental phase, when overall sprint ability is determined by physical growth, muscle strength plays a crucial role in modulating the spatiotemporal characteristics of sprinting. However, the overall explanatory power of the SF model was relatively low (Adjusted *R* ^*2*^ = 0.128), suggesting that other unmeasured factors also played a role in determining the SF during this period. Similarly, MVC was a significant predictor of shorter CT. However, this finding must be interpreted with caution as the overall model for CT did not reach statistical significance (*P* = 0.129).

### 4.4 Determinants of Maximal Sprint Speed in the Post‐BP Period

Multiple regression analysis of the post‐BP group revealed that LL was not a significant predictor of MSS. However, LL remained a significant predictor of all spatiotemporal variables; it was a positive predictor of SL, CT and FT and a negative predictor of SF (Table 1). These results suggested that LL played a crucial role in mediating the interaction between SL and SF, whereas its direct effect on MSS appeared to be minimal. Previous studies have indicated that an increase in LL provides a geometric advantage for greater SL but may also lead to an increase in the moment of inertia of the lower limb, resulting in greater mechanical demands on leg recovery (Ropret et al. 1998; Hunter et al. 2004). Indeed, previous research reported a negative relationship between LL and SF (Miyashiro et al. 2019), supporting the present findings in the post‐BP group. This suggests that although LL directly determines sprinting ability via SL before BP, its role shifts after BP to influence the interaction between SL and SF, making it no longer a direct determinant of sprinting ability.

By contrast, the influence of MVC and MT on sprinting ability was more pronounced in the post‐BP group. Multiple regression analysis demonstrated that MVC and MT were significant positive predictors of MSS (Table 1). Furthermore, MVC and MT were significant positive predictors of SF, whereas MT was a significant negative predictor of CT (Table 1). This is likely because increased maximal strength and muscle mass allow for greater ground reaction force (GRF) during the support phase of sprinting. Specifically, a greater vertical GRF can simultaneously result in shorter CT and higher SF (Weyand et al. 2010).

Taken together, these results highlight a shift in the determinants of sprinting ability, from a reliance on anthropometric growth in the pre‐BP phase to a dominance of neuromuscular factors in the post‐BP phase.

### 4.5 Practical Implications

The findings of this study provide important insights for designing sprint training programs for children and for refining talent identification processes. First, the determinants of sprint ability differed between the children before and after +1.1 years of MO. Specifically, before +1.1 years of MO, anthropometric factors, such as LL, exhibited a greater influence on sprint ability than on muscle strength. In this pre‐BP phase, given that LL is strongly associated with maturity (Figure 4), it is reasonable to assume that more mature children exhibit greater LL and superior sprinting abilities than less mature children within this specific group. However, because individual differences in LL exist even among children of the same chronological age and maturity status, it is essential to consider both maturity status and LL in the talent identification process for athletes in the pre‐BP period.

In contrast, after +1.1 years of MO, sprinting ability is less influenced by maturity level and LL. Consequently, direct comparison of sprinting ability among children at this developmental stage may be more appropriate without heavily considering their maturity status. This shift in the determinants of sprinting ability also has implications for the optimisation of sprint training interventions. Although strength training is beneficial for long‐term athletic development at all stages, our findings suggest that its direct impact on sprinting ability improvement may become more pronounced after BP. In the pre‐BP phase, in which performance gains are strongly linked to anthropometric growth, training can prioritise the development of motor skills and coordination to help athletes effectively manage their growth. Subsequently, in the post‐BP phase, as sprint speed becomes more dependent on neuromuscular factors, targeted strength and power training are likely to become a more direct stimulus for enhancing sprint performance.

### 4.6 Limitations

This study has certain limitations. First, although our maturity estimation utilised retrospective longitudinal height data, the analysis of the performance variables was cross‐sectional. Therefore, the developmental trends observed in this study require confirmation through fully longitudinal studies that track both maturation and performance over time. Second, our categorisation of participants into pre‐ and post‐BP groups based on a single point (+1.1 years of MO) did not account for the error term associated with MO estimation, meaning that some individuals near the BP may have been misclassified. Although this approach preserved our sample size, the potential impact of this misclassification should be considered when interpreting group comparison results. Third, potential multicollinearity was observed in the regression models for the pre‐BP group as indicated by the elevated VIF values for LL (5.81) and MVC (4.87). This suggests a moderate correlation between these predictors, which likely reflects the natural tendency of both limb length and muscle strength to increase concurrently during this phase of maturation. Consequently, the individual contributions of LL and MVC to the pre‐BP models should be interpreted with caution. Finally, some key findings may have been specific to this sample. For instance, the negative correlation between MO and LL observed in the post‐BP group (Figure 4). Biologically, body height and limb length are generally expected to increase or remain stable as maturation progresses beyond PHV; however, our data showed no such positive correlation between MO and Height as well (Figure S1, *r* = 0.081 and *P* = 0.582). This result may reflect a recruitment bias in the post‐BP group, where more mature individuals happened to have shorter stature or shorter limbs. This trend likely influenced the observed stagnation in MSS, and its generalisability warrants caution. Both this finding and the timing of BP itself (+1.1 years of MO) should be reexamined in other samples with longitudinal designs to validate their applicability to a wider population.

## 5 Conclusions

This study aimed to clarify the developmental process of sprinting ability and spatiotemporal variables in developing children aged 7.6–17.9 years and to identify the determining factors from the perspective of muscle strength and leg length. Our results demonstrated that BP in the developmental trend of sprinting ability occurred at 1.1 years after PHV. Anthropometric factors, particularly LL, had a significant effect on SL and MSS before BP. In contrast, after BP, strength‐related components, such as MVC and MT, influenced SF and MSS in addition to anthropometric variables. These findings indicate that the determinants of sprinting ability shifted by approximately 1.1 years after PHV.

## Author Contributions

**Masamichi Okudaira:** data curation, investigation, writing – original draft. **Ryosuke Takeda:** Data curation, investigation, writing – review and editing. **Tetsuya Hirono:** data curation, investigation, writing – review and editing. **Taichi Nishikawa:** data curation, investigation, writing – review and editing. **Shun Kunugi:** data curation, investigation, writing – review and editing. **Kaito Igawa:** data curation, investigation, writing – review and editing. **Saeko Ueda:** data curation, investigation, writing – review and editing. **Yukiko Mita:** supervision, project administration, writing – review and editing. **Kohei Watanabe:** conceptualisation, supervision, project administration, writing – review and editing.

## Funding

This study was financially supported by a Grant‐in‐Aid for Research Activity Start‐up (22K21254) from the Japan Society for the Promotion of Science to MO.

## Conflicts of Interest

The authors declare no conflicts of interest.

## Supporting information


**Figure S1**: Relationship between height, body mass and maturity offset (MO) in the pre‐BP group (open circles) and the post‐BP group (closed circles). A and B represent height and body mass. The vertical dotted line represents +1.1 years of MO (breakpoint in maximal sprint speed development). The solid line represents the regression line when correlations were significant within groups, and the shaded area represents the 95% confidence interval.

## Data Availability

Data that support the findings of the present study are presented in the text, figures and table and are available from the corresponding author upon reasonable request.

## References

[ejsc70133-bib-0001] Beunen, G. , and R. M. Malina . 1988. “Growth and Physical Performance Relative to the Timing of the Adolescent Spurt.” Exercise and Sport Sciences Reviews 16, no. 1: 503–540. 10.1249/00003677-198800160-00018. 3292266

[ejsc70133-bib-0002] Dorn, T. W. , A. G. Schache , and M. G. Pandy . 2012. “Muscular Strategy Shift in Human Running: Dependence of Running Speed on Hip and Ankle Muscle Performance.” Journal of Experimental Biology 215, no. Pt 11: 1944–1956. 10.1242/jeb.064527. 22573774

[ejsc70133-bib-0003] Hunter, J. P. , R. N. Marshall , and P. J. Mcnair . 2004. “Interaction of Step Length and Step Rate During Sprint Running.” Medicine & Science in Sports & Exercise 36, no. 2: 261–271. 10.1249/01.Mss.0000113664.15777.53. 14767249

[ejsc70133-bib-0004] McKay, A. K. , T. Stellingwerff , E. S. Smith , et al. 2022. “Defining Training and Performance Caliber: A Participant Classification Framework.” International Journal of Sports Physiology and Performance 17, no. 2: 317–331. 10.1123/ijspp.2021-0451. 34965513

[ejsc70133-bib-0005] Meyers, R. W. , J. L. Oliver , M. G. Hughes , J. B. Cronin , and R. S. Lloyd . 2015. “Maximal Sprint Speed in Boys of Increasing Maturity.” Pediatric Exercise Science 27, no. 1: 85–94. 10.1123/pes.2013-0096. 25054903

[ejsc70133-bib-0006] Mirwald, R. L. , A. D. Baxter‐Jones , D. A. Bailey , and G. P. Beunen . 2002. “An Assessment of Maturity From Anthropometric Measurements.” Medcine and Science in Sports Exercise 34, no. 4: 689–694. 10.1097/00005768-200204000-00020. 11932580

[ejsc70133-bib-0007] Miyashiro, K. , R. Nagahara , K. Yamamoto , and T. Nishijima . 2019. “Kinematics of Maximal Speed Sprinting With Different Running Speed, Leg Length, and Step Characteristics.” Frontiers in Sports and Active Living 1: 37. 10.3389/fspor.2019.00037. 33344960 PMC7739839

[ejsc70133-bib-0008] Muggeo, V. M. 2003. “Estimating Regression Models With Unknown Break‐Points.” Statistics in Medicine 22, no. 19: 3055–3071. 10.1002/sim.1545. 12973787

[ejsc70133-bib-0009] Murayama, R. , K. Kigoshi , and K. Sugiura . 2023. “Development of a Method for Predicting the Maturity Offset for Peak Height Velocity Suitable for Japanese Youth.” International Journal of Sport and Health Science 21: 1–8. 10.5432/ijshs.202206.

[ejsc70133-bib-0010] Myrvang, S. , and R. van den Tillaar . 2024. “The Longitudinal Effects of Resisted and Assisted Sprint Training on Sprint Kinematics, Acceleration, and Maximum Velocity: A Systematic Review and Meta‐Analysis.” Sports Medcine – Open 10, no. 1: 110. 10.1186/s40798-024-00777-7. PMC11469994 39392558

[ejsc70133-bib-0011] Nagahara, R. , M. Haramura , Y. Takai , et al. 2019. “Age‐Related Differences in Kinematics and Kinetics of Sprinting in Young Female.” Scandinavian Journal of Medicine & Science in Sports 29, no. 6: 800–807. 10.1111/sms.13397. 30697820

[ejsc70133-bib-0012] Nagahara, R. , T. Matsubayashi , A. Matsuo , and K. Zushi . 2014a. “Kinematics of Transition During Human Accelerated Sprinting.” Biology Open 3, no. 8: 689–699. 10.1242/bio.20148284. 24996923 PMC4133722

[ejsc70133-bib-0013] Nagahara, R. , and M. Murata . 2020. “Inertial Measurement Unit Based Hip Flexion Strength‐Power Test for Sprinters.” Frontiers in Sports and Active Living 2: 571523. 10.3389/fspor.2020.571523. 33345132 PMC7739800

[ejsc70133-bib-0014] Nagahara, R. , H. Naito , J. B. Morin , and K. Zushi . 2014b. “Association of Acceleration With Spatiotemporal Variables in Maximal Sprinting.” International Journal of Sports Medicine 35, no. 9: 755–761. 10.1055/s-0033-1363252. 24577864

[ejsc70133-bib-0015] Nagahara, R. , Y. Takai , M. Haramura , et al. 2018a. “Age‐Related Differences in Spatiotemporal Variables and Ground Reaction Forces During Sprinting in Boys.” Pediatric Exercise Science 30, no. 3: 335–344. 10.1123/pes.2017-0058. 29478372

[ejsc70133-bib-0016] Nagahara, R. , Y. Takai , H. Kanehisa , and T. Fukunaga . 2018b. “Vertical Impulse as a Determinant of Combination of Step Length and Frequency During Sprinting.” International Journal of Sports Medicine 39, no. 4: 282–290. 10.1055/s-0043-122739. 29415292

[ejsc70133-bib-0017] Naimo, M. A. , A. N. Varanoske , J. M. Hughes , and S. M. Pasiakos . 2021. “Skeletal Muscle Quality: A Biomarker for Assessing Physical Performance Capabilities in Young Populations.” Frontiers in Physiology 12: 706699. 10.3389/fphys.2021.706699. 34421645 PMC8376973

[ejsc70133-bib-0018] Oliver, J. L. , R. S. Lloyd , and M. C. Rumpf . 2013. “Developing Speed Throughout Childhood and Adolescence: The Role of Growth, Maturation and Training.” Strength and Conditioning Journal 35, no. 3: 42–48. 10.1519/SSC.0b013e3182919d32.

[ejsc70133-bib-0019] Philippaerts, R. M. , R. Vaeyens , M. Janssens , et al. 2006. “The Relationship Between Peak Height Velocity and Physical Performance in Youth Soccer Players.” Journal of Sports Science 24, no. 3: 221–230. 10.1080/02640410500189371. 16368632

[ejsc70133-bib-0020] Ropret, R. , M. Kukolj , D. Ugarkovic , D. Matavulj , and S. Jaric . 1998. “Effects of Arm and Leg Loading on Sprint Performance.” European Journal of Applied Physiology and Occupational Physiology 77, no. 6: 547–550. 10.1007/s004210050374. 9650741

[ejsc70133-bib-0021] Rumpf, M. C. , J. B. Cronin , J. Oliver , and M. Hughes . 2015. “Kinematics and Kinetics of Maximum Running Speed in Youth Across Maturity.” Pediatric Exercise Science 27, no. 2: 277–284. 10.1123/pes.2014-0064. 25389204

[ejsc70133-bib-0022] Salo, A. I. , I. N. Bezodis , A. M. Batterham , and D. G. Kerwin . 2011. “Elite Sprinting: Are Athletes Individually Step‐Frequency or Step‐Length Reliant?” Medicine & Science in Sports & Exercise 43, no. 6: 1055–1062. 10.1249/MSS.0b013e318201f6f8. 20980924

[ejsc70133-bib-0023] Schepens, B. , P. A. Willems , and G. A. Cavagna . 1998. “The Mechanics of Running in Children.” Journal of Physiology 509, no. Pt 3: 927–940. 10.1111/j.1469-7793.1998.927bm.x. 9596810 PMC2231007

[ejsc70133-bib-0024] Stricker, P. R. , A. D. Faigenbaum , T. M. McCambridge , Council on Sports Medicine and Fitness , M. A. Brooks , G. Canty , A. B. Diamond , W. Hennrikus , K. Logan , K. Moffatt , B. A. Nemeth , K. B. Pengel , and A. R. Peterson . 2020. “Resistance Training for Children and Adolescents.” Pediatrics 145, no. 6: e20201011. 10.1542/peds.2020-1011. 32457216

[ejsc70133-bib-0025] Towlson, C. , S. Cobley , G. Parkin , and R. Lovell . 2018. “When Does the Influence of Maturation on Anthropometric and Physical Fitness Characteristics Increase and Subside?” Scandinavian Journal of Medicine & Science in Sports 28, no. 8: 1946–1955. 10.1111/sms.13198. 29668045

[ejsc70133-bib-0026] Weyand, P. G. , R. F. Sandell , D. N. Prime , and M. W. Bundle . 2010. “The Biological Limits to Running Speed are Imposed From the Ground up.” Journal of Applied Physiology (1985) 108, no. 4: 950–961. 10.1152/japplphysiol.00947.2009. 20093666

