# Supplementary material for: Determinants of Sprint Ability Change During Maturation in Developing Children
Source: Eur J Sport Sci. 2026 Jan 30;26(2):e70133. doi: 10.1002/ejsc.70133 (PMC12856720; doi:10.1002/ejsc.70133)
Supplement: Supplementary file 1 — Figure S1: Relationship between height, body mass and maturity offset (MO) in the pre‐BP group (open circles) and the post‐BP group (closed circles). A and B represent height and body mass. The vertical dotted line represents +1.1 years of MO (breakpoint in maximal sprint speed development). The solid line represents the regression line when correlations were significant within groups, and the shaded area represents the 95% confidence interval. [file EJSC-26-e70133-s001.docx]

**Supplemental Figure 1.**

Relationship between height, body mass, maturity offset (MO) in the pre-BP group (open circles) and the post-BP group (closed circles). A and B represent height and body mass. The vertical dotted line represents +1.1 years of MO (breakpoint in maximal sprint speed development). The solid line represents the regression line when correlations were significant within groups, and the shaded area represents the 95% confidence interval.
